# Supplementary figures and images for: Activation of specific mushroom body output neurons inhibits proboscis extension and sucrose consumption
Source: PLoS One. 2020 Jan 28;15(1):e0223034. doi: 10.1371/journal.pone.0223034 (PMC6986700; doi:10.1371/journal.pone.0223034)

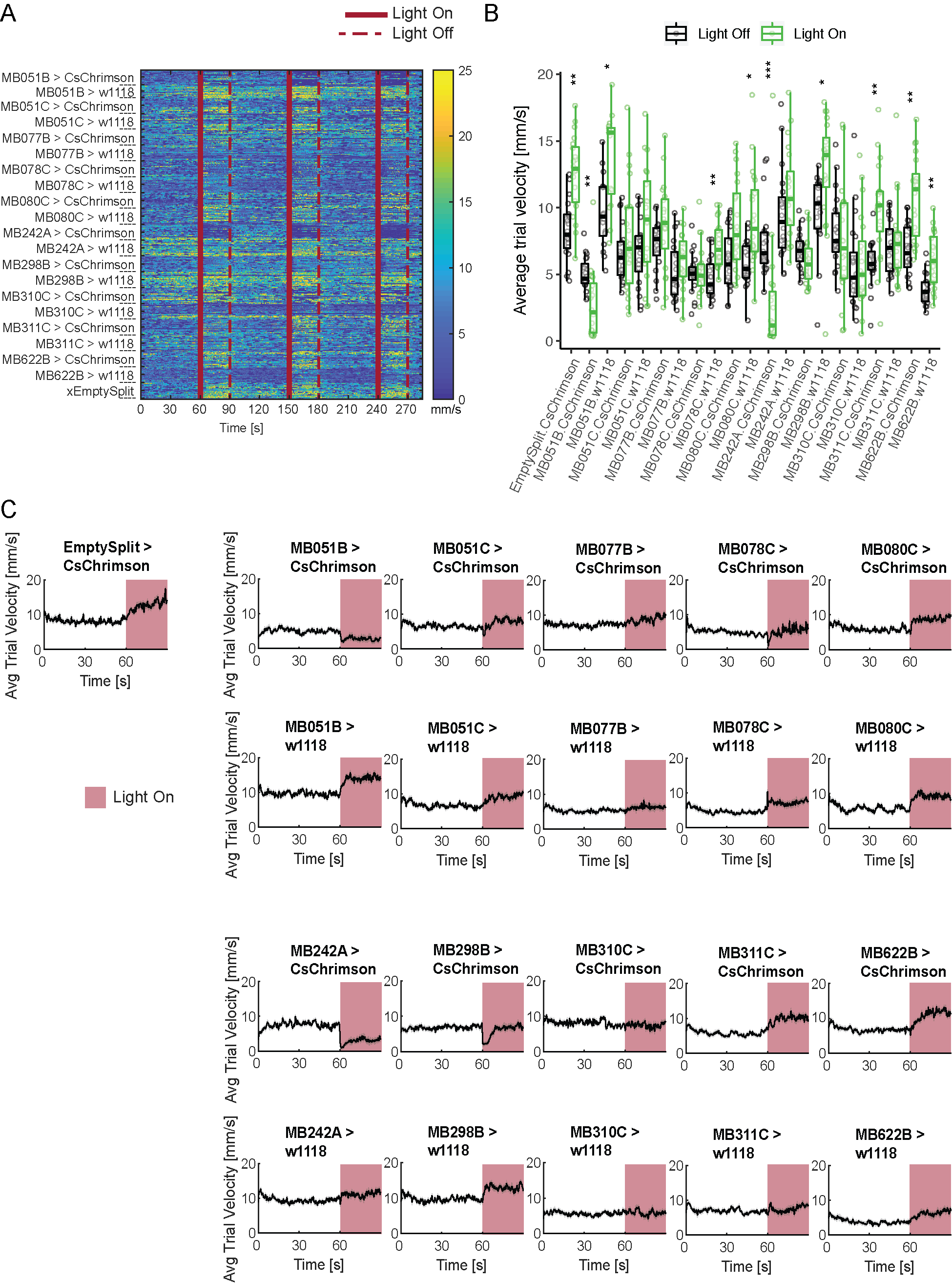

Supplement: S1 Fig — Single MBON split-Gal4, UAS-CsChrimson flies were placed in a circular agar bowl arena. Fly position was tracked under IR illumination with a camera, and then subsequently analyzed using ctrax and custom matlab scripts. The stimulation protocol was 3x (60s off, 30s on pulsing 633nm light at 50 Hz), and a total of 5 minutes of video was recorded for each trial. A) Velocity heat map, each row is an individual fly. Genotypes are denoted. B) Box and whiskers plot of average velocity over 3 trials, for light off and light on periods. Each data point is one fly, whiskers = 10th to 90th percentile, box = 25th to 75th percentile, and line in box = median. Statistical significance was calculated using unpaired Wilcoxon Rank Sum tests with Bonferroni correction, *p<0.05, **p<0.01, ***p<0.001. C) Bounded line plots. For each fly, the average velocity over 3 trials of (60s Light Off, 30s Light On) was calculated. The black line represents the mean average trial velocities of n = 16–20 flies for each genotype; the shaded grey areas represent the standard error. MBON split-Gal4 lines crossed with UAS-CsChrimson are on top, paired with the genetic controls of MBON split-Gal4 lines crossed with w1118, bottom. The red line marks the beginning of the light on period at 60s. (TIF) [file pone.0223034.s001.tif]

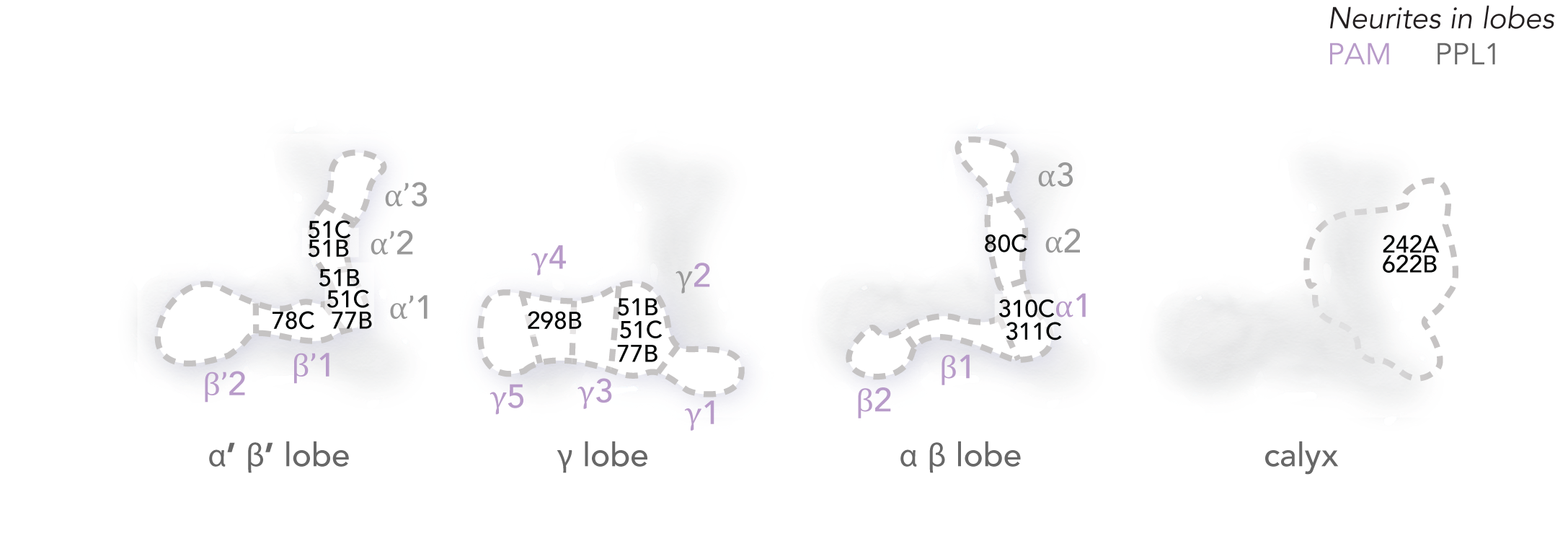

Supplement: S2 Fig — Each lobe of the MB, as well as the calyx, is drawn separately for visual clarity. The name of each MBON split-Gal4 is spatially localized to the compartments where it has dendritic arborizations. Colors indicate cluster of origin for DANs. (TIF) [file pone.0223034.s002.tif]

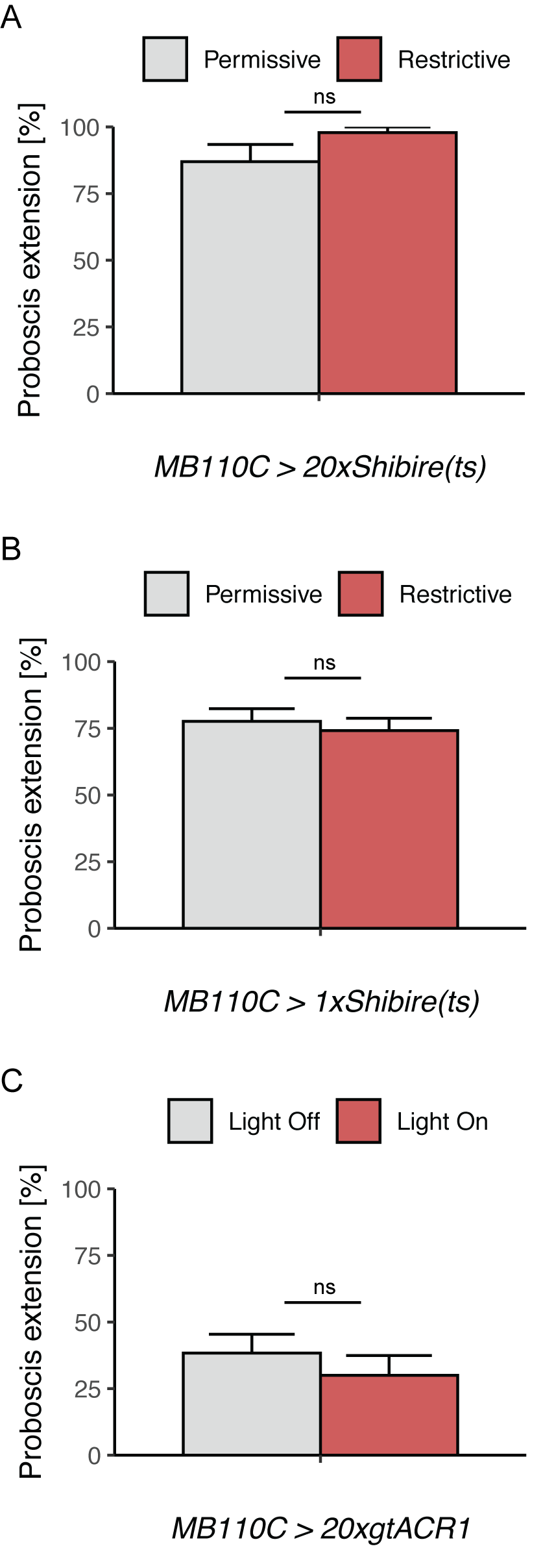

Supplement: S3 Fig — A) MB110C was conditionally silenced with 20xShibirets and PER to tarsal sugar presentation (100 mM sucrose) was recorded. Silencing with this method did not change PER rate. n = 23. Permissive temperature = 20–22°C, restrictive = 30–32°C. B) MB110C was conditionally silenced with 1xShibirets and PER to 50 mM sucrose on the legs was recorded. Silencing MBONs with this method did not change PER rate. n = 58. C) MB110C was silenced acutely with the light-gated anion channelrhodopsin 20xgtACR1 and PER to 10 mM sucrose on the legs was recorded. Silencing MBONs with this method did not result in a change in PER rate. n = 58. For all graphs, error bars indicate mean ± SEM. Statistical significance was determined by Wilcoxon Rank Sum tests, ns = not significant. (TIF) [file pone.0223034.s003.tif]

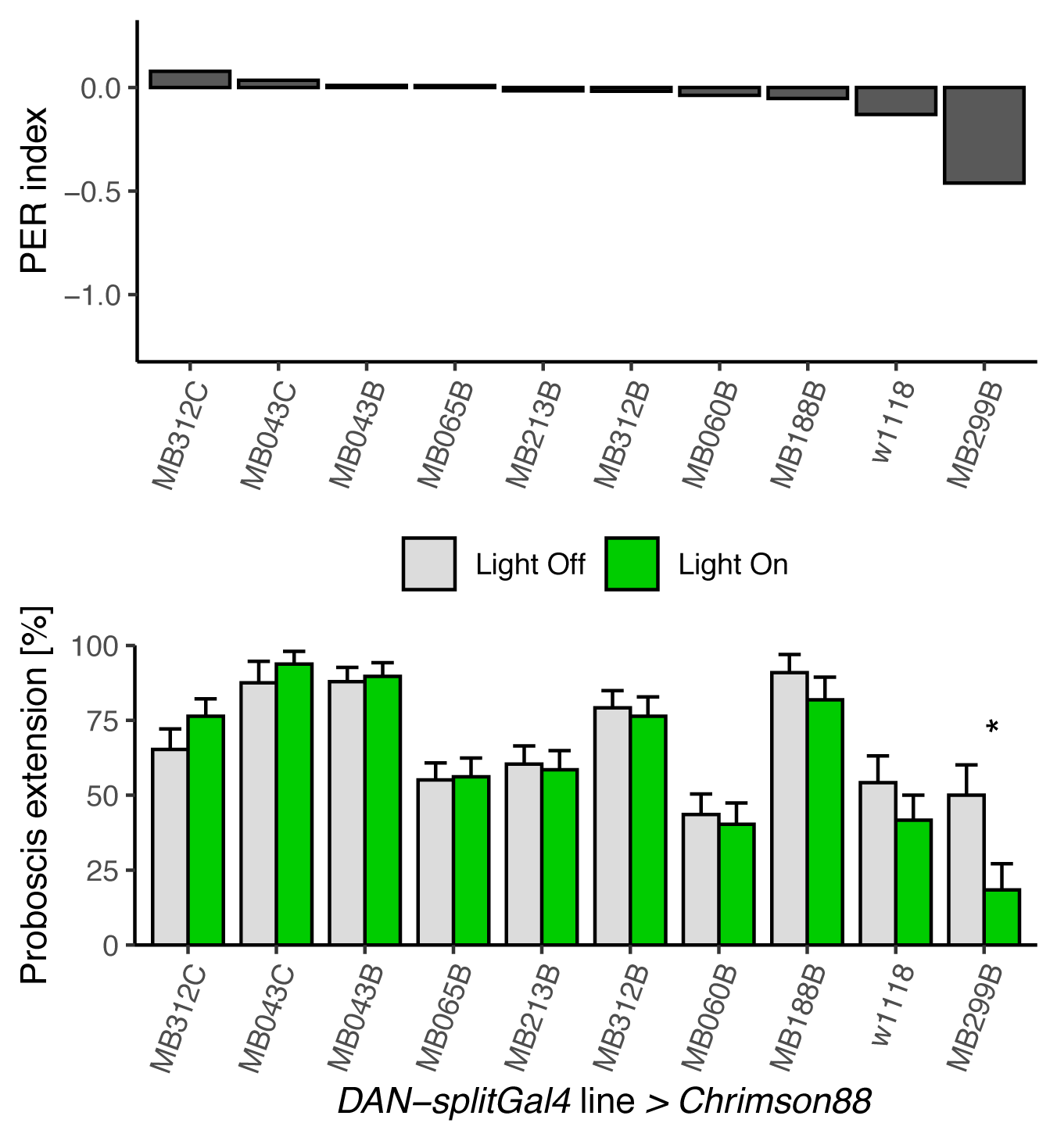

Supplement: S4 Fig — Behavioral screen for flies that change proboscis extension rate when DANs are activated. DAN split-Gal4 lines were crossed to UAS-Chrimson88 for light induced activation and tested for proboscis extension to 50 mM sucrose presentation to the tarsi, and then simultaneous sucrose presentation to the tarsi and red laser light. Extension rates were compared between light and dark conditions in the same fly (n = 19–53 flies per line). Values represent mean ± SEM. Statistical significance was calculated using paired Wilcoxon Rank Sum tests (light versus no light) with Bonferroni correction, *p < 0.05. (TIF) [file pone.0223034.s004.tif]

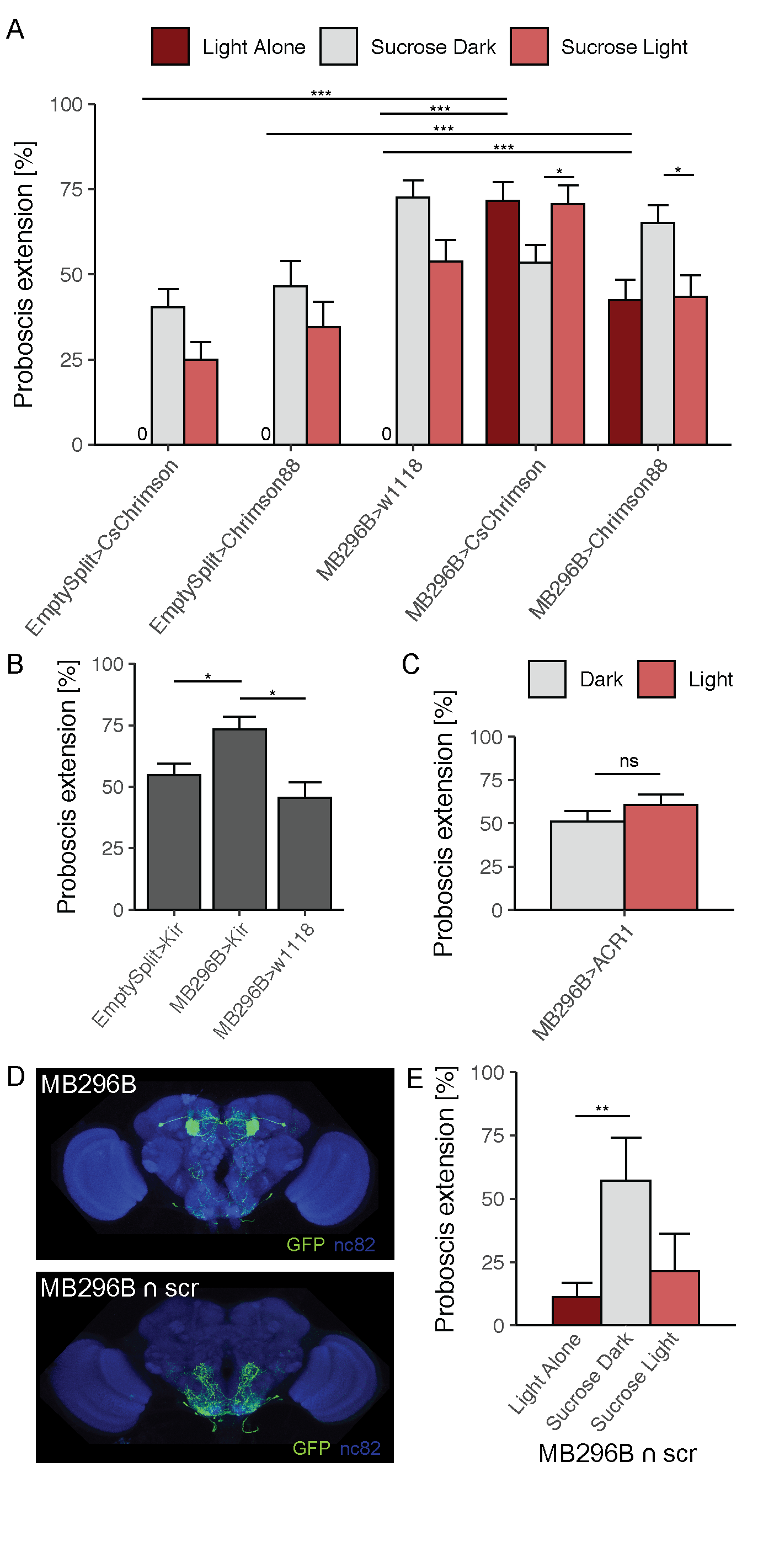

Supplement: S5 Fig — A) MB296B split-Gal4 was crossed to UAS-Chrimson88 and UAS-CsChrimson for light induced activation, and tested for proboscis extension in 3 conditions: (1) red light alone, (2) 30 mM sucrose to the tarsi, and (3) simultaneous red light and sucrose presentation to the tarsi. Extension rates were compared between each condition in the same fly and between different fly genotypes of the same condition for genetic controls (n = 27–58 flies). Values represent mean ± SEM. Statistical significance was calculated using paired Wilcoxon Rank Sum tests (same flies, different conditions) or unpaired Wilcoxon Rank Sum tests (flies of different genotypes, same treatment condition) with Bonferroni correction, *p < 0.05, ***p < 0.001. Green bars represent flies given sucrose and red light. Grey bars represent flies given sucrose. B) MB296B was inhibited with Kir2.1 and PER to 30 mM sucrose on the legs was recorded. Silencing with Kir2.1 increased PER (n = 44–56, Mean ± SEM). Statistical significance was determined by Wilcoxon Rank Sum tests with Bonferroni correction, *p<0.05. C) Candidates were silenced with 20xgtACR1 and PER to 30 mM sucrose on the legs was recorded, in the absence and presence of green light. n = 47, mean ± SEM. Statistical significance was determined by a paired Wilcoxon test. D) Top: Projection pattern of MB296B. Bottom: Projection pattern of the SEZ neuron labeled by MB296B, as determined by an intersection between the Hox gene scr and MB296B. E) MB296B-split-Gal4 was crossed to 20xUAS-dsFRT-CsChrimson.mVenus; LexAop-FLP/CyO; scr-LexA/TM2 flies for light induced activation, and tested for proboscis extension to light (n = 31.) Of these 31 flies, 7 were also tested for their responses to 30 mM sucrose to the tarsi, and simultaneous red light and sucrose presentation to the tarsi as well. Statistical significance was calculated using Wilcoxon Rank Sum tests, **p<0.01. (TIF) [file pone.0223034.s005.tif]
